# A Systematic Review of Economic Evaluations of Chemoprophylaxis for Tuberculosis

**DOI:** 10.1155/2011/130976

**Published:** 2011-11-01

**Authors:** Shraddha Chavan, David Newlands, Cairns Smith

**Affiliations:** ^1^Population Health Section, Institute of Applied Health Sciences, University of Aberdeen, Aberdeen AB24 3fx, UK; ^2^Economics Department, Business School, University of Aberdeen, Aberdeen AB24 3fx, UK

## Abstract

Since treatment of active disease remains the priority for tuberculosis control, donors and governments need to be convinced that investing resources in chemoprophylaxis provides health benefits and is good value for money. The limited evidence of cost effectiveness has often been presented in a fragmentary and inconsistent fashion. *Objective*. This review is aimed at critically reviewing the evidence of cost effectiveness of chemoprophylaxis against tuberculosis, identifying the important knowledge gaps and the current issues which confront policy makers. *Methods*. A systematic search on economic evaluations for chemoprophylaxis against tuberculosis was carried out, and the selected studies were checked for quality assessment against a standard checklist. *Results*. The review provides evidence of the cost effectiveness of chemoprophylaxis for all age groups which suggests that current policy should be amended to include a focus on older adults. Seven of the eight selected studies were undertaken wholly in high income countries but there are considerable doubts about the transferability of the findings of the selected studies to low and middle income countries which have the greatest incidence of latent tuberculosis infection. *Conclusion*. There is a pressing need to expand the evidence base to low and middle income countries where the vast majority of sufferers from tuberculosis live.

## 1. Background

Tuberculosis (TB) is a leading cause of morbidity and mortality worldwide. Two million people a year die of tuberculosis, making it the single leading microbial killer of adults [1]. The vast majority of cases occur in low and middle income countries. In 2008, the WHO regions of Europe and the Americas accounted for only 8% of the global number of incident cases [2].

A further threat is latent tuberculosis infection (LTBI) in which there is the risk of developing active disease. The current recommended standard chemoprophylaxis therapy for LTBI prevention is 9 months of isoniazid (9INH) which has an efficacy of more than 90% if taken properly [3, 4]. Isoniazid preventive therapy (IPT) has a greater protective effect on childhood TB, reducing the chance of developing probable or definite TB by 72% [5].

Since treatment of active disease remains the priority for TB control, donors and governments need to be convinced that investing resources in chemoprophylaxis provides health benefits and is good value for money. However, most studies focus on high income settings, and there is uncertainty of whether similar effects can be expected in low and middle income countries where the severity of the problem is different. There is accumulated evidence of the individual [6, 7] and public health [8] benefits of chemoprophylaxis in high income settings, but very few studies of its cost effectiveness. The limited evidence of the cost effectiveness of preventive measures against TB is often presented in a fragmentary and inconsistent fashion [9]. This review is aimed at critically reviewing the evidence of cost effectiveness of chemoprophylaxis against TB, identifying the important knowledge gaps and the current issues which confront policy makers.

## 2. Methods

### 2.1. Literature Search

A systematic search on economic evaluations for chemoprophylaxis against TB was carried out in July 2010, by the first named author, on the following databases: EMBASE (1980–2010), using keywords “economic evaluation,” “cost,” “cost-effectiveness,” “chemoprophylaxis,” and “tuberculosis”; NHS EED, HRD, and HEED (CRD database: http://www.crd.york.ac.uk/crdweb/), using keywords “tuberculosis,” “prevention,” and “cost”; MEDLINE (1950–2010), using 30 different search terms. The bibliographies of available papers were checked for any additional relevant studies.

### 2.2. Inclusion Criteria

Studies eligible for inclusion were economic evaluations of chemoprophylaxis against TB. There were no restrictions on the drug type or duration of therapy. The intervention could be directly observed treatment, which is under supervision, or self-administered treatment. There were no restrictions on the age and sex of study populations or on the types of study design, cost analysis, and perspective of economic evaluations. Non-English studies were excluded.

The outcomes included (when available) were the incremental cost-effectiveness ratio (ICER) for cost per life year saved, number of TB cases and TB related deaths, number needed to treat (NNT) to prevent one TB case, number of drug-induced hepatic toxicity incidents, the endpoint of withdrawals due to toxicity, and lack of patient compliance.

**Figure 1:**
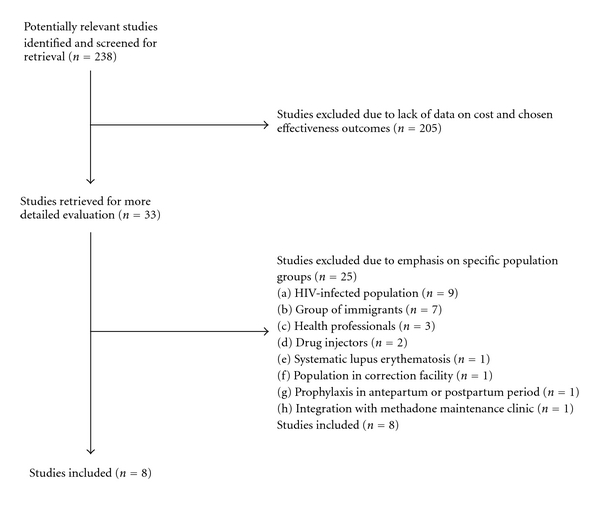
Flow chart of study selection process.

Searches for economic evidence on chemoprophylaxis against TB yielded a total of 238 published articles. After exclusion and hand searches, 33 economic studies relevant to the review topic were identified. However, many of these studies were concerned with specific groups such as HIV-positive people, immigrants, drug injectors, prisoners, and health professionals. Since results derived from such studies cannot be applied to the general population, they were excluded from the final selection. Although this review did not include economic and societal benefits of prevention of TB in HIV patients, countries with high incidence of HIV and TB, use of IPT along with ART should be highly cost effective. The study selection process is summarized in Figure 1. 

Eight economic evaluation studies of chemoprophylaxis against TB [11–18] were included in this systematic review, summarized in Table 1.

### 2.3. Quality Assessment

The selected studies were assessed against a standard checklist of best practice in economic evaluation. This checklist consists of ten questions covering such issues as evidence of effectiveness, the careful measurement and valuation of costs and consequences, and allowance for uncertainty [19]. The scoring tool is similar to QUADAS and other such methodology checklists. Studies were scored 1 for each “yes” answer, 0 for “no,” and 0.5 for “partially.” Initial scoring was done by the first two named authors independently of one another with almost complete agreement. Subsequent discussion led to the swift achievement of total consensus. The eight studies are scored against this checklist in Table 2. 

## 3. Results

### 3.1. Result of Quality Assessment

All bar two, Fitzgerald and Gafni [17] and Ziakas and Mylonakis [18], scored very well, with a total of 9 or 9.5 out of 10. 

 In addition, although the final question of the checklist concerns “issues of concern to users” of the economic evaluation results, it is increasingly common to separately consider the ease of transferability of the results to other contexts [20]. Transferability depends on similarities in demographic and epidemiological characteristics but also the health system structures necessary to deliver treatment effectively. Transferability is likely therefore to be strong between high income contexts like Germany, the US, and Canada, countries covered in the selected studies, but not from high income contexts to low and middle income countries. Indeed, all the selected studies performed equally well in terms of their transferability to other high income countries, apart from Tan et al. [16]. This study is based on data on subgroups from British Columbia which would be difficult to transfer to other settings.

 In summary, five studies [7, 11–15] scored well, both in terms of the best practice checklist and the transferability of results to other (high income) countries.

### 3.2. Cost-Effectiveness Results

Isoniazid dominates no intervention, which means it costs less and provides greater health benefits, for all groups [7, 12–14] or high risk groups [11]. In turn, two studies find that rifampin dominates isoniazid [15, 18].

 Only three studies presented incremental cost-effectiveness ratio (ICER) results but none are comparable, one being the ICER of isoniazid over no intervention, for low risk groups [11], the second rifampin over no intervention [7, 13], and the third isoniazid-rifapentine over rifampin [15]. However, for what it is worth, the ICER values reported are all reasonable, implying that each first named (more expensive) treatment is cost effective. Meta-analysis was not considered because of the heterogeneity of the studies. The effectiveness measures differ among studies, as do the currency and cost year.

## 4. Discussion

### 4.1. Age

Due to increased risk of isoniazid induced hepatotoxicity, American Thoracic Society in 1974 restricted the use of isoniazid prophylaxis to low-risk tuberculin reactors older than 35 years of age. But revised 1983 guidelines recommended clinicians to evaluate and monitor liver function and discontinue treatment if aminotransferase levels exceed 3–5 times normal values [3]. The majority of the selected studies found evidence of the cost effectiveness of chemoprophylaxis for people over 35-year old which suggests that current policy should be amended to include a focus on older adults. A further study [21] confirmed the health benefits of INH prophylaxis to all age groups, apart from those aged over 80. Such results might help practitioners to implement current guidelines without fear of hepatotoxicity. 

Among children, Tan et al. [16] found that direct implementation of preventive treatment of non-BCG-vaccinated household contacts of less than 10-year old is justified.

### 4.2. Different Drug Regimens for Prophylaxis

Holland et al. [15] found that the 4-month rifampin approach was associated with significant lifelong cost savings for most programs in the US and Canada although the results did not include information on compliance and hepatotoxicity. Jasmer et al. [13] show that, in spite of the shorter duration of the 2-month treatment with rifampin-pyrazinamide (RZ), the associated high cost of routine laboratory monitoring for significant higher-risk hepatotoxicity and hospitalization made it less cost effective than 6-month isoniazid treatment. Regarding 4-month rifampin, one study [22] found that patient acceptance and compliance is excellent but argued for further research on its safety and efficacy. Ziakas and Mylonakis [18] also found evidence of the superiority of 4-month Rifampin compared to 9-month INH therapy in terms of safety, compliance, and cost.

### 4.3. Drug-Induced Hepatotoxicity

A 1983 revision of guidelines issued by the American Thoracic Society recommended clinicians to evaluate and monitor liver function and discontinue treatment if aminotransferase levels exceed 3–5 times normal values [23]. Two studies of monitored prophylaxis based upon these revised guidelines [24, 25] found very low mortality rates, of about 0.001%, similar to the rate of death from acute hepatitis and acute liver necrosis from any cause in the general population. Salpeter et al. [12] conclude that monitored isoniazid prophylaxis should be extended to tuberculin reactors of all ages.

### 4.4. Transferability

Seven of the eight selected studies were undertaken in high income countries: four in the US, two in Canada, and one in Germany. All these studies used cost data and life tables from country databases. The eighth study was a pooled meta-analysis which used US cost data and patient-related data from eight Canadian centers, two US centers, one Saudi Arabian centre, and one Brazilian centre. 

Clearly, the selected studies have most relevance to high income countries, precisely those which have the lowest burden of TB. However, the results, particularly those that relate to cost effectiveness, are difficult to transfer to different settings. The level and structure of costs of treatment of LTBI differ considerably across the world. The prices of drugs, which are internationally traded, will not generally vary much, but prices of local goods and services, such as the salaries of health professionals or transport costs, can vary enormously. While the prices of drugs may not differ much, their availability and quality can vary. There may be considerable economies of scale. The cost per TB case prevented will often diminish rapidly as the number of cases prevented increases—assuming that the appropriate infrastructure is in place to treat a greater number of cases [26].

 While the greatest differences may lie with such economic factors, countries also vary with regard to their organizational structures which influence their capacity to screen, treat, and monitor patients on a regular basis [27]. More broadly, countries differ in their demographic and epidemiological characteristics and in treatment compliance rates. As a result of all these factors, there are considerable doubts about the transferability of the findings of the eight selected studies to low and middle income countries which have the greatest incidence of LTBI.

### 4.5. Research and Policy Implications

The eight selected studies, particularly the five which score well on all measures of quality assessment [11–15], provide robust evidence of the effectiveness and cost effectiveness of preventive TB treatment in high income countries. However, there is an urgent research need to produce evidence for low and middle income countries. For example, not even the World Health Organisation (WHO) provides comprehensive guidelines for preventive therapy [28]. It would be a very slow and costly process to replicate the type of careful analyses conducted in the selected studies in this paper in every country in which decisions have to be made about the allocation of scarce health care resources to TB treatment and prevention. Instead, population and decision modeling techniques can be employed. WHO-CHOICE, developed by the World Health Organization (WHO), is an attempt to provide relevant information on health effects and cost structures at a regional level which can then be adapted to the country level by the use of national data [29].

 In policy terms, the priority use of scarce resources in some low and middle income countries may still be treatment of active rather than latent cases of TB. However, for those countries which are able to expand their preventive programmes, the implications of the selected studies for policy and practice are summarized in Table 1. For high income countries at least, the current recommendation of use of isoniazid for 9 months is cost effective (as compared to no prophylaxis). It yields significant health and cost benefits. However, policy makers need to consider expanding treatment to cover all age groups, so long as therapy is monitored. Monitoring during therapy increases the cost of care in the short run. But it leads to better compliance and a reduction in secondary transmission with consequent benefits in long term.

 In global terms, growing evidence of the effectiveness and cost effectiveness of preventive treatment of TB could help mobilize donor funding [10] to invest in the development of evidence for a programme for chemoprophylaxis in low and middle income countries.

## 5. Conclusion

The studies selected in this paper provide clear evidence of the health benefits and cost effectiveness of chemoprophylaxis for TB, but this evidence is derived almost totally from three high income countries: the US, Canada, and Germany. There are great variations in terms of economic factors, organisational structure, and epidemiological characteristics between nonendemic and endemic countries. In the former, the transmission of disease is well controlled and the chance of experiencing TB infection is lower. The impact of preventive therapy in low risk settings cannot be extrapolated to TB endemic countries where the effect of preventive therapy could be transient. We argue that there is a pressing need to expand the evidence base to low and middle income countries where the vast majority of sufferers from TB live. The fight against TB calls for an increase emphasis on prevention for the most vulnerable groups. 

## Figures and Tables

**Table:** (a)

	Study objective	Study population and setting
Rose et al. [11]	To compare TB prevention with isoniazid (INH) chemoprophylaxis to no intervention, for low risk as well as high-risk tuberculin reactors	Men aged 20, recently infected with tubercle bacillus and thus at high risk; men aged 55, older tuberculin reactors having low risk of activation US

Salpeter et al. [12]	To evaluate the effectiveness and cost effectiveness of monitored INH prophylaxis for low-risk tuberculin reactors older than 35 years of age	35, 50 and 70-year-old low risk tuberculin reactors who have normal chest radiograph and are not at increased risk of tuberculin activation US

Jasmer et al. [13]	To determine cost effectiveness of rifampin-pyrazinamide (RZ) for 2 months compared with INH for 6 months for treatment of latent tuberculosis in adults without HIV infection	Adult aged 17 years or older with a tuberculin skin test result, in whom active TB is excluded and in whom treatment of latent TB infection would ordinarily be recommended; exclusion criteria are pregnancy, HIV infection, and history of gout US

Diel et al. [14]	To perform a cost-effectiveness analysis in young-and middle-aged adults with latent tuberculosis infection	20- and 40-year old close contacts of active TB cases with positive Mantoux test and in whom active TB is excluded Germany

Holland et al. [15]	To evaluate cost and cost effectiveness of different regimens for treatment of LTBI	Hypothetical cohort of individuals with LTBI contacts of infectious case, all adults with average age of 39 years US

Tan et al. [16]	To evaluate cost effectiveness of LTBI therapy for different TB contact population defined by important risk factors and to propose optimal policy based on different recommendations for each subgroup of contact	TB contacts with tuberculin test size ≥5 mm, defined by age group (<10 y/o or above), ethnicity (Canadian born/foreign born), BCG vaccination status British Columbia, Canada

Fitzgerald and Gafni [17]	To evaluate role of INH prophylaxis in low-risk patients with positive Mantoux test result and identify most efficient use of health care resources	20-, 50-, and 70-years-old low-risk patients with positive Mantoux test Canada

Ziakas and Mylonakis [18]	To compare efficacy, toxicity, and cost of the 4-month Rifampin treatment (4RIF) with the standard 9-month INH strategy (9INH) from pooled meta-analysis of published clinical studies	Patient-related data from eight Canadian centres, two US centres, one Saudi Arabian centre, and one Brazilian centre

**Table:** (b)

	Measure of outcomes	Cost effectiveness	Implications
Rose et al. [11]	Life years gained, quality-adjusted life years (QALYs) Incremental cost effectiveness ratio (ICER)	For high-risk reactors over 35, isoniazid dominates no intervention; cost savings and greater benefits (increased life expectancy). For low-risk reactors over 35, ICER of isoniazid over no intervention of $12,625 per year of life gained and $35,011 per death averted	Study can contribute to a change in existing policy and practice; consideration of INH therapy for all infected persons irrespective of age group and risk of tuberculin reactors

Salpeter et al. [12]	Number needed to treat, life years gained, and probability of survival at 1-year Cost saving	Isoniazid dominates no intervention for 35, 50 and 70 year olds; cost savings and increased life expectancy	Study can contribute to a change in existing policy and practice; consideration of all age groups for preventive therapy leading to potential public health benefits

Jasmer et al. [13]	Number of TB cases averted, number of TB-related deaths ICER	Isoniazid dominates no intervention; cost savings and increased life expectancy, more deaths prevented Isoniazid costs less than rifampin-pyrazinamide; both treatments have the same gain in life expectancy ICER of rifampin over no intervention of $2,494 per case prevented	Justify existing policy of INH prophylaxis

Diel et al. [14]	Number needed to treat, number of TB-related deaths avoided Cost saving	Isoniazid dominates no intervention for 20 and 40 year olds; cost savings, more cases, and TB-related deaths prevented	Acceleration of expansion of INH prevention

Holland et al. [15]	Life years gained, QALYs ICER	Rifampin dominates (self-administered and, directly observed) isoniazid; cost savings and more QALYs gained, more cases of active TB-prevented ICER of isoniazid-rifapentine over rifampinof $48,999 per QALY gained	Study can contribute to a change in existing policy and practice; highlights important knowledge gaps

Tan et al. [16]	Number of active TB cases prevented, QALYs Net monetary benefit	Test and treat (with isoniazid) more cost effective (in terms of net monetary benefit, the difference between benefits, valued at $50,000 per QALY, and costs) than no screening and treat all, for most subgroups	Justifies existing policy; support current practice of provision of treatment on the basis of TST size; exclusion of low-risk groups from screening and providing treatment to high-risk contacts without screening could improve the performance of the program

Fitzgerald and Gafni [17]	Number of TB cases prevented, life years gained Direct as well as indirect costs in different age groups	Average cost per case prevented in low-risk patients by isoniazid of $8,586 (20-year old), $28,260 (50 year old), and $40,102 (70-year old)	Justifies existing policy of INH prophylaxis; considers of all age groups; highlights importance of including indirect as well as direct costs

Ziakas and Mylonakis [18]	Hepatotoxicity, compliance Cost saving	Rifampin dominates isoniazid; cost savings and lower risk of noncompletion, lower rate of hepatotoxicity	Justifies existing policy; 9INH therapy is considered as standard of care

**Table 2:** Checklist of best practice in economic evaluation studies.

	Rose	Salpeter	Jasmer	Diel	Holland	Fitzgerald	Tan	Ziakas
(1) Was a well-defined question posed in answerable form?	1	1	1	1	1	0.5	1	1
(2) Was a comprehensive description of the competing alternatives given?	1	1	1	1	1	1	1	1
(3) Was the effectiveness of the programme or services established?	1	1	1	1	1	1	1	1
(4) Were all the important and relevant costs and consequences for each alternative identified?	0.5	0.5	0.5	1	1	0.5	1	0.5
(5) Were costs and consequences measured accurately in appropriate physical units?	1	1	1	1	1	1	1	0.5
(6) Were the costs and consequences valued credibly?	1	1	0.5	1	1	0.5	1	1
(7) Were costs and consequences adjusted for differential timing?	1	1	1	1	1	0.5	1	0.5
(8) Was an incremental analysis of costs and consequences of alternatives performed?	1	1	1	1	1	0	1	1
(9) Was allowance made for uncertainty in the estimates of costs and consequences?	1	1	1	1	1	1	1	1
(10) Did the presentation and discussion of results include all issues of concern to users?	0.5	0.5	1	0.5	0.5	0.5	0.5	0.5

Total	9	9	9	9.5	9.5	6.5	9.5	8
